# Identifying risk factors and mortality rate of premature coronary artery disease in young Saudi population

**DOI:** 10.1038/s41598-024-62970-8

**Published:** 2024-06-03

**Authors:** Thamir Al-Khlaiwi, Syed Shahid Habib, Nervana Bayoumy, Huthayfah Al-Khliwi, Sultan Ayoub Meo

**Affiliations:** 1 Department of Physiology, College of Medicine, King Saud University, Riyadh, Saudi Arabia; 2 College of Medicine, King Saud University, Riyadh, Saudi Arabia

**Keywords:** Premature coronary artery disease, Risk factors, Diabetes mellitus, Dyslipidemia, Hypertension, Smoking, Physiology, Cardiology, Health care, Risk factors

## Abstract

Coronary artery disease is a leading cause of morbidity and mortality worldwide. It occurs due to a combination of genetics, lifestyle, and environmental factors. Premature coronary artery disease (PCAD) is a neglected clinical entity despite the rising number of cases worldwide. This study aimed to investigate the risk factors of premature coronary artery disease. In this study, we searched articles that had studied the risk factors of premature coronary artery diseases from January 2000 to July 2022 in Saudi Arabia in Web of Science, Pub Med, Scopus, Springer, and Wiley databases. The final analysis is based on seven articles. The smoking prevalence was 39%, diabetes mellitus 41%, hypertension 33%, overweight and obesity 18%, family history of coronary artery disease (CAD) 19%, dyslipidemia 37%, and the prevalence range of low-density lipoprotein cholesterol was 33.8–55.0%. The results revealed a mortality prevalence of 4% ranging from 2 to 8% which is similar to the prevalence in older patients which was 2–10%. Smoking, diabetes mellitus, hypertension, family history of CAD, dyslipidemia, and overweight/obesity are significantly and positively associated with premature coronary artery diseases. The health authorities should design and implement an intensive and effective prophylactic plan to minimize the subsequent impact of PCAD on the young population. In addition, early diagnosis of PCAD has great value in providing timely treatment, managing the patients, and minimizing the burden of the disease.

## Introduction

An alarming estimate by health organizations and authorities shows that cardiovascular diseases (CVD) might be the leading cause of death worldwide in 2030^1,2^. Around one-third of the mortality rate globally in 2020 was due to cardiovascular diseases^3,4^. Although Coronary artery diseases (CAD) are expected to occur more frequently in the elderly population, recent reports highlighted that 10% of patients less than 45 years old are also affected by CAD^5^. This is called Premature coronary artery disease (PCAD) which occurs below the age of 45 to 55 years^6,7^.

In North America, an increase in the incidence of Myocardial infarction (MI) was observed in younger patients recently^8,9^. The increase in the incidence of myocardial infarction was the consequence of predisposing risk factors leading to premature coronary artery diseases. Some of these risk factors are common with coronary artery disease (CAD) affecting the elderly populations as well as premature coronary artery disease while others are not^10^.

Several traditional risk factors have been identified to be associated with premature coronary artery diseases including smoking, physical inactivity, overweight/obesity, metabolic disorders, diabetes mellitus, and hypertension while multiple nontraditional risk factors including heterozygous familial hypercholesterolemia (HeFH), inflammation, prothrombotic states and ethnicity^11^. Early detection and identification of risk factors are of great value in reducing the impact of premature coronary artery diseases and their consequences.

Worldwide, CAD is the most common cause of mortality and loss of Disability Adjusted Life Years (DALYs), and the burden falls in low and middle-income countries. CAD caused about 7 million deaths and 129 million DALYs annually^12^. In 2015 CAD accounted for 8.9 million deaths and 164.0 million DALYs. PCAD is a leading public health challenge resulting in years of productive life loss and an intensifying burden on health systems^13,14^.

In Saudi Arabia, it has been found that admitted patients with acute coronary syndrome (ACS) were 10 years younger than similar patients in other countries and were mostly diabetic or hypertensive^15–17^. We aimed in this systematic review and meta-analysis to evaluate the prevalence of various premature coronary artery disease risk factors in Saudi Arabia. This systematic review and meta-analysis will help health authorities design prophylactic strategies against a hidden threat to protect the younger population.

## Methods

### Data search strategy

The studies were searched from the Web of Science, PubMed, Scopus, Springer, and Wiley databases from January 2000 to July 2022. The specific keywords have been used in the search strategy in different combinations: “coronary artery disease,” “coronary heart disease,” “vascular disease,” “atherosclerosis,” “smoking”, “diabetes mellitus”, “risk factors,” and “Saudi Arabia.” We assigned two co-authors to evaluate the literature by excluding irrelevant articles by reading the titles and abstracts. In addition, if any unclear abstracts correlated to our objectives, the full article was retrieved and studied thoroughly. After exclusion, the remaining relevant articles were evaluated by another co-author for final eligibility.

### Inclusion and exclusion criteria

Any Studies that evaluate patients with PCAD or compare PCAD to healthy subjects in Saudi Arabia. The cut-off age of Premature coronary disease is different according to various studies but mostly mention less than 45 and 55 years^6,7^. All articles published were in the English language. The duplicate studies, brief communications, case reports, letters to editors, and systematic reviews, were excluded from the study.

### Data extraction

Information from the eligible articles, after filtration, was arranged in a table (Table 1). The information from each article was: first author, sample size, age of patients, study design, and outcomes. The prevalence of traditional and nontraditional was extracted from eligible studies including smoking, hypertension, diabetes mellitus, dyslipidemia, family history of CAD, obesity, and drug abuse.

**Table 1:** Descriptive characteristics of selected studies with outcome (N = 7).

Refs.	Region	Participants	Study design	Age	Outcome
Al-murayeh et.al. ^18^	Southern region	157	Case–control	Males < 45 Females < 55	Acute coronary syndrome (ACS)
Al-Shahrani et al. ^19^	Eastern region	652 patients. 109, patients < 45	Retrospective case–control study	< 45	Acute coronary syndrome (ACS)
Al Khadra ^20^	Eastern region	65	Retrospective study	< 45	Acute myocardial infarction
Al ghamdi et al. ^21^	Central region	22	Retrospective study	< 40	Acute coronary syndrome (ACS)
Sakr et al. ^22^	Central region	A total of 402 patients. 197 patients < 45	Retrospective case–control study	< 45	ST-segment elevation myocardial infarction (STEMI)
Abazid et al. ^23^	Central region	159	Cross-sectional	< 55	Coronary artery calcification
Al-saif et al. ^24^	Multiple regions	5055 patients. 2275 patients < 55	Prospective multi-hospital registry	< 55	Acute coronary syndrome (ACS)

### Quality assessment of the studies

The Newcastle–Ottawa Scale (NOS), Table 2, which assesses selection bias, comparability of exposed and control participants, and outcome evaluation, was used to evaluate the quality of non-randomized research. Each criterion was given a star rating of 1 or 0 stars. For observational studies, the overall star rating ranged from 0 to 9 stars. The NOS instrument assesses three areas: study group selection (maximum 4 stars); study group comparability (maximum 2 stars), and outcome assessment (max 3 stars). The quality was evaluated independently by two authors, and disagreements were settled by discussion. A study with a score of 7–9 or 10 is good quality, a score of 4–6 is fair quality, and a score of 0–3 is poor quality.

**Table 2:** Newcastle–Ottawa quality assessment scale for cohort studies.

Study	Selection				Design or analysis controlled for confounders	Outcome			Quality score
	Representativeness of the case-cohort	Selection of the control cohort	Ascertainment of outcome	Demonstration that outcome of interest was not present at the start of the study		Assessment of the outcome	Follow-up was long enough for outcomes to occur	Adequacy of follow-up of cohorts	
Al-Murayeh et al. ^18^	★	★	★	0	★	★	0	0	6 (Fair)
Al-Shahrani et al. ^19^	★	★	★	0	★	★	0	0	6 (Fair)
Al-Khadra, ^20^	★	0	★	0	★	★	0	0	4 (Fair)
Alghamdi et al. ^21^	★	0	★	0	★	★	0	0	4 (Fair)
Sakr et al. ^22^	★	★	★	0	★	★	0	0	6 (Fair)
Abazid et al. ^23^	★	★	★	0	★	★	0	0	6 (Fair)
Al-Saif et al. ^24^	★	★	★	0	★	★	0	0	6 (Fair)

Table 2 summarizes the quality assessment scores for the included studies. Five studies scored three stars. For the remaining studies, the reasons for not receiving a full quality score for the selection section were that there was no demonstration that the outcome of interest was not present at the start of the study or that the control group was not selected from the general community. Five studies controlled the outcomes and for additional factors (e.g., age), so they scored two stars, while 2 studies controlled the outcomes and scored only one star. All studies reported the ascertainment of the outcome, but they did not describe the follow-up period, so they scored only one star.

### Statistical analysis

The statistical evaluations were carried out utilizing the Comprehensive Meta-Analysis version 3 (Biostat Inc. USA). The prevalence of different risk factors was calculated along with a 95% confidence interval. The significance level was established at a *p*-value less than 0.05. The Cochrane chi-squared test was used to assess article heterogeneity; a *P* value of 0.05 or higher indicates the presence of heterogeneity. I^2^ value was calculated to determine the effect of heterogeneity on the meta-analysis. The pooled studies did exhibit moderate to high levels of heterogeneity, as indicated by I^2^ values > 50% and *P *< 0.05. If I^2 ^< 50% and *P *> 0.05, a fixed-effects design was used; otherwise, a random-effects design was utilized. To evaluate the robustness of this meta-analysis, a sensitivity analysis was conducted. Egger’s test was conducted to evaluate publication bias. This latter was further assessed by the visual inspection of the symmetry in funnel plots.

## Results

**Figure 1:**
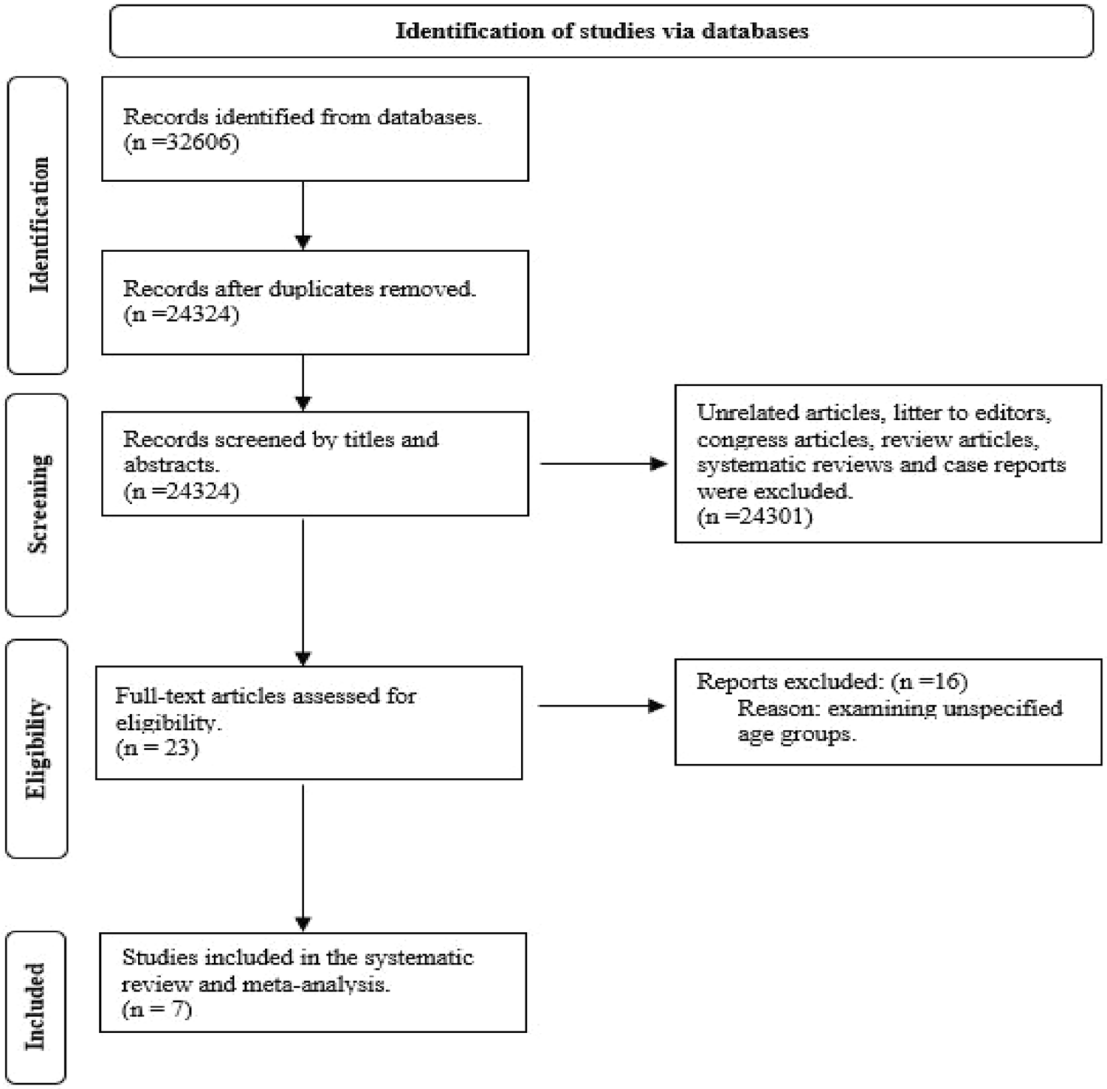
Flow chart of the study design according to PRISMA guidelines.

Initial 32606 articles were screened. 32583 articles, then, were excluded by reading the title and abstract. The excluded studies involved the following: Unrelated articles, litter to editors, congress articles, review articles, systematic reviews, and case reports. The remaining 23 full‐text articles were further examined in depth for eligibility. Finally, 7 articles were eligible to be included. Figure 1 shows the flow chart that was followed for eligibility according to the PRISMA guidelines^17^. Table 1 illustrates the characteristics of the studies that were included in the systematic review; two of the studies were retrospective case-control studies, two retrospective studies, two case-control studies, and one cross-sectional study. All the studies were from patients presenting to hospitals^18–24^.

### Prevalence of risk factors

The prevalence range percentage of risk factors are shown in table 3. Seven studies mentioned smoking as a risk factor for PCAD in Saudi Arabia^18–24^. The range of smoking prevalence was from 29 to 52%.

**Table 3:** Summary of prevalence for risk factors of premature coronary artery disease in eligible studies (N = 7).

Risk factors	Prevalence range percentage (%)
Smoking	29–52
Diabetes	34–49
Hypertension	24–45
Overweight/obesity	5–62
Family history of CAD	7–49
Drug abuse	3.0–5.8
Dyslipidemia	22–60
LDL	33.8–55.0
HDL	52.8
Hypertriglyceridemia	7.7

Seven studies mentioned DM as a risk factor for PCAD in Saudi Arabia^18–24^. The range of DM prevalence was from 34 to 49%.

Seven studies mentioned hypertension as a risk factor for PCAD in Saudi Arabia. The range of hypertension prevalence was from 24 to 45%. One study reported hypertension prevalence of patients less than 40 years was 28%^24^.

Two studies reported overweight and obesity as risk factors for PCAD in Saudi Arabia^18,22^. The range of overweight and obesity prevalence was from 5 to 62%.

Four studies reported a family history of PCAD as a risk factor for PCAD in Saudi Arabia^19,20,22,23^. The family history of CAD prevalence ranged from 7 to 49%.

Two studies reported Drug abuse and Amphetamine-type stimulants (ATS) as a risk factor for PCAD in Saudi Arabia^21,22^. One study mentioned drug abuse in general as a risk factor with a prevalence of 3.0%^22^ while another study specified Amphetamine-type stimulants (ATS) as a risk factor with a prevalence of 5.8%^21^.

The different studies have mentioned various cutoff points in defining hypercholesterolemia. levels of total cholesterol > 5.2 mmol/L and > 6.2 mmol/L were used as a cutoff point^25,26^.

Four studies reported dyslipidemia in general as a risk factor for PCAD in Saudi Arabia^18,22–24^. The range of dyslipidemia prevalence was from 22 to 60%. One study reported dyslipidemia prevalence in patients less than 40 years was 32% and from 41 to 55 years was 39%^24^.

Two studies reported increased Low‐density lipoprotein cholesterol (LDL) as a risk factor for PCAD in Saudi Arabia^19,20^. The range of Low‐density lipoprotein cholesterol (LDL) prevalence was from 33.8 to 55.0%. Only one study mentioned decreased High‐density lipoprotein cholesterol (HDL) as a risk factor for PCAD in Saudi Arabia^20^. The prevalence was 52.8%. One study mentioned Hypertriglyceridemia as a risk factor for PCAD in Saudi Arabia^20^, and the prevalence was 7.7%. The present study results showed a mortality prevalence ranging from 0 to 9.2% which is similar to the prevalence in older patients from 2 to 10%^18–24^.

### Smoking

**Figure 2:**
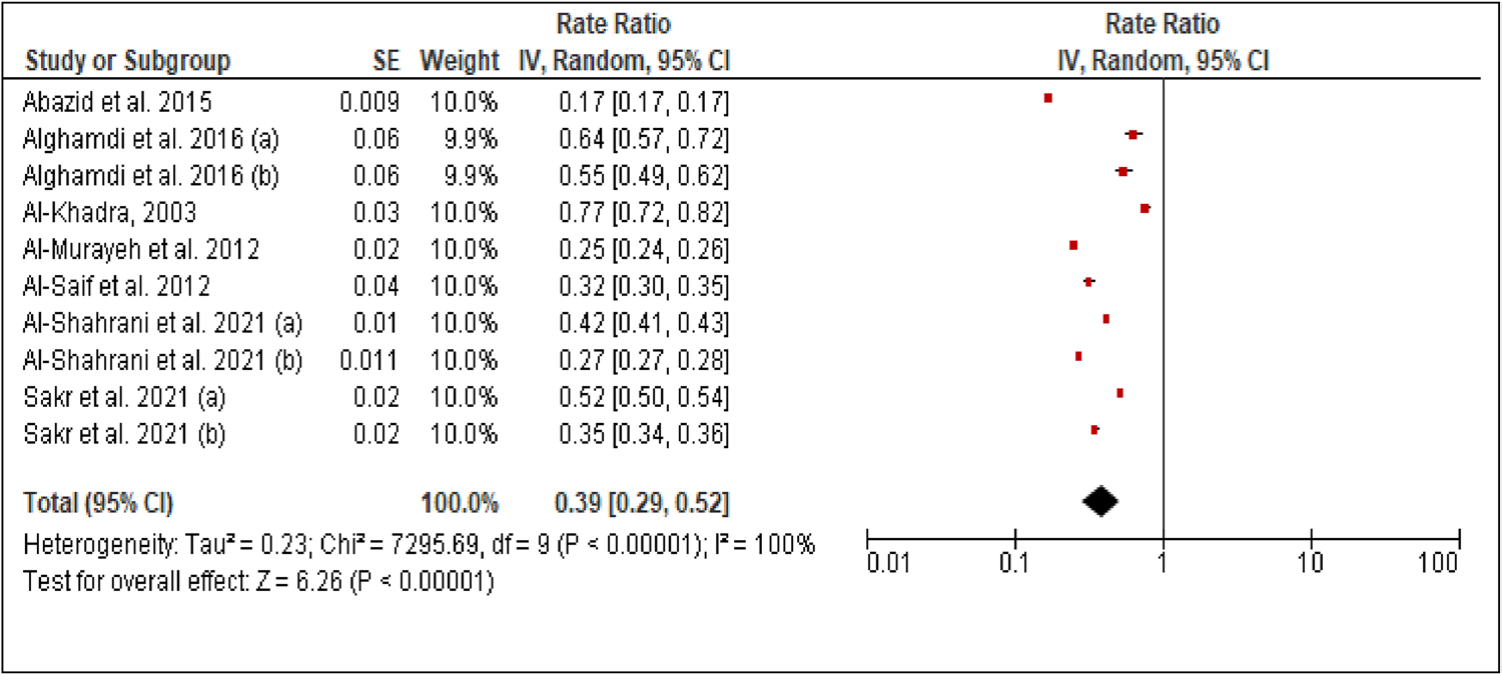
Forest plot of the prevalence of smoking among patients with PCAD. (**a**): under 45 years. (**b**): above 45 years.

All studies reported the prevalence of smoking among patients with PCAD. The heterogeneity (Chi^2^ = 7295.69, *p *< 0.00001, I^2^= 100%) was high, so a random effect model was used. The forest plot analysis showed a pooled estimated prevalence of 39% (95% CI 29–52%) (Figure 2).

### Diabetes mellitus

**Figure 3:**
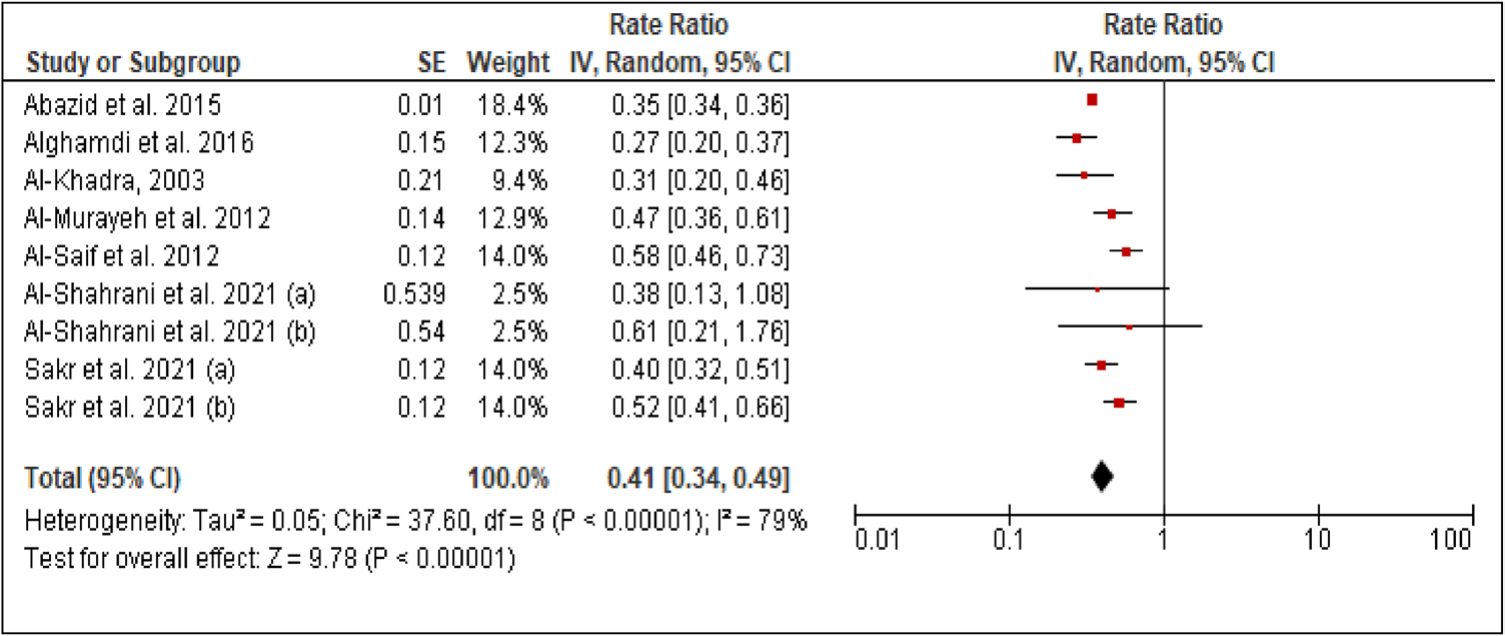
Forest plot of the prevalence of DM among patients with PCAD. (**a**): under 45 years (**b**): above 45 years.

All studies reported the prevalence of DM among patients with PCAD. The heterogeneity (Chi^2^ = 37.60, *P *< 0.00001, I^2 ^= 79%) was high, so a random effect model was used. The forest plot analysis showed a pooled estimated prevalence of 41% (95% CI 34–49%) (Figure 3).

### Hypertension

**Figure 4:**
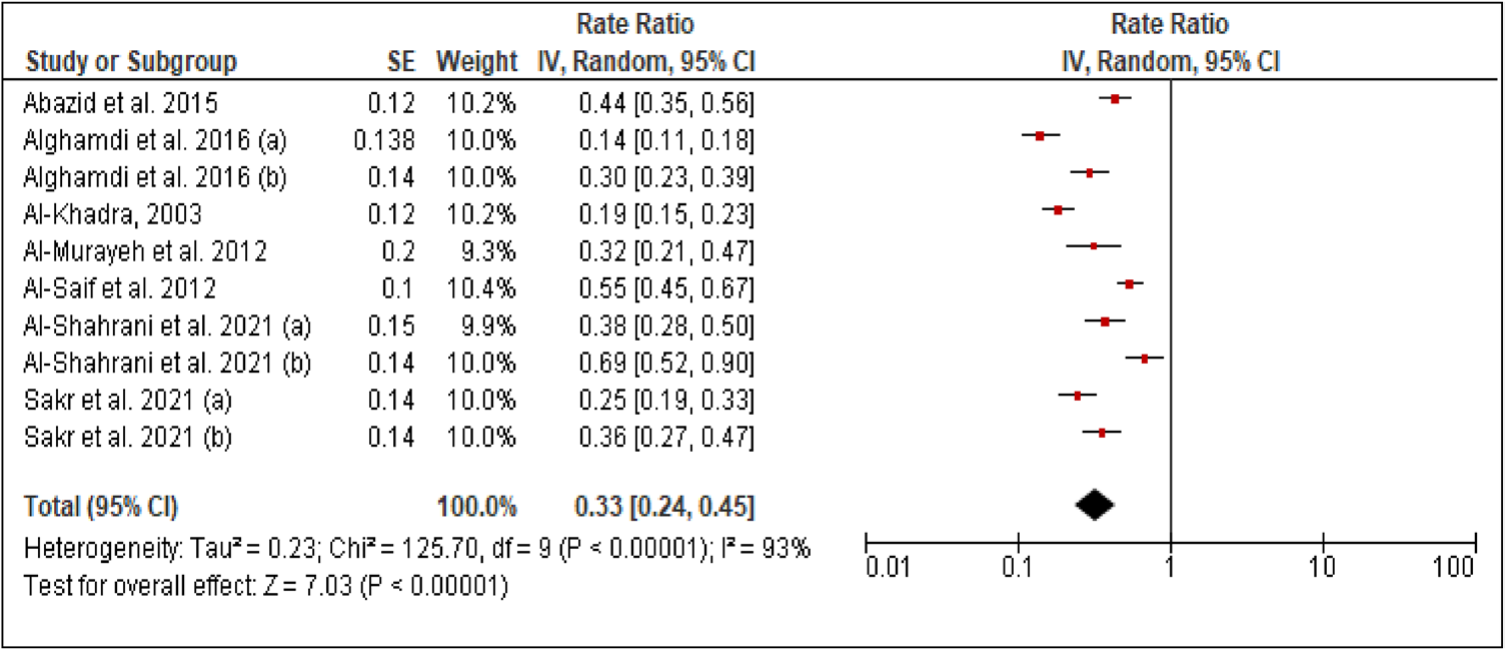
Forest plot of the prevalence of hypertension among patients with PCAD. (**a**): under 45 years; (**b**): over 45 years.

All studies reported the prevalence of hypertension among patients with PCAD. The heterogeneity (Chi^2 ^= 125.70, *p *< 0.0001, I^2 ^= 93%) was high, so a random effect model was used. The forest plot analysis showed a pooled estimated prevalence of 33% (95% CI 24–45%) (Figure 4).

### Overweight and obesity

**Figure 5:**
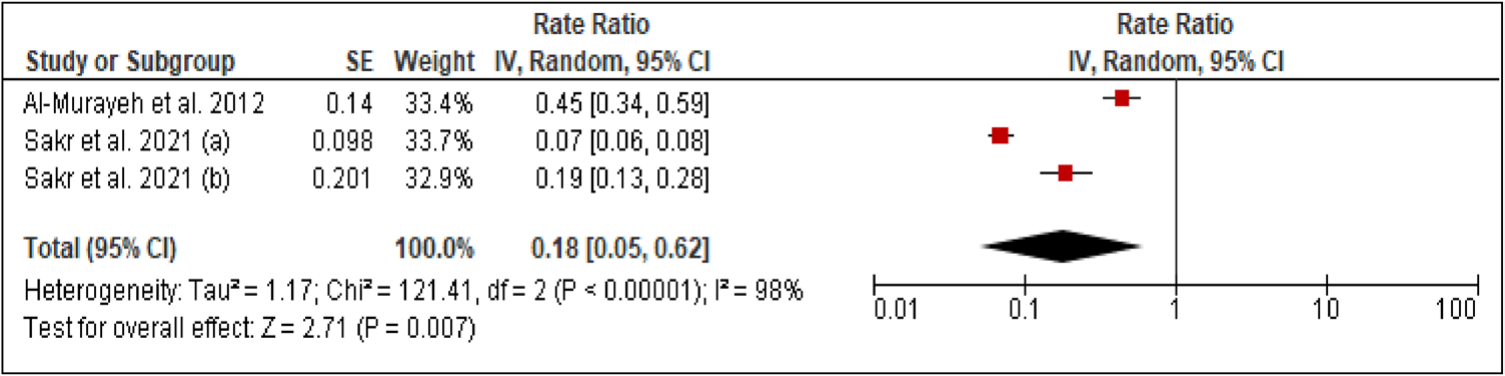
Forest plot of the prevalence of overweight and obesity among patients with PCAD. (**a**): under 45 years; (**b**): above 45 years.

Two studies reported the prevalence of overweight and obesity among patients with PCAD. The heterogeneity (Chi^2 ^= 121.41, *p *< 0.00001, I^2 ^= 98%) was high, so a random effect model was used. The forest plot analysis showed a pooled estimated prevalence of 18% (95% CI 5–62%) (Figure 5).

### Family history of coronary artery disease (CAD)

**Figure 6:**
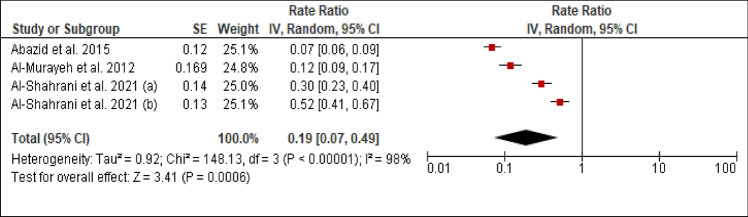
Forest plot of the prevalence of family history of CAD among patients with PCAD. (**a**): under 45 years; (**b**): above 45 years.

Three studies reported the prevalence of family history of CAD among patients with PCAD. The heterogeneity (Chi^2 ^= 148.13, *p *< 0.00001, I^2 ^= 98%) was high, so a random effect model was used. The forest plot analysis showed a pooled estimated prevalence of 19% (95% CI 7–49%) (Figure 6).

### Dyslipidemia

**Figure 7:**
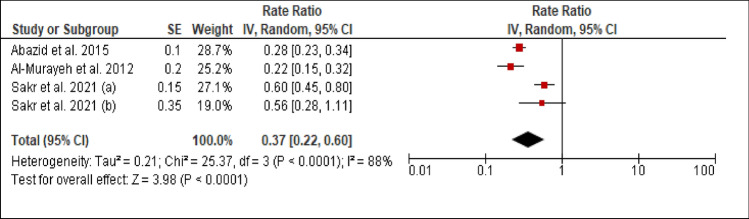
Forest plot of the prevalence of dyslipidemia among patients with PCAD, (**a**): under 45 years; (**b**): above 45 years.

Three studies reported the prevalence of dyslipidemia among patients with PCAD. The heterogeneity (Chi^2 ^= 25.37, *p *< 0.00001, I^2 ^= 88%) was high, so a random effect model was used. The forest plot analysis showed a pooled estimated prevalence of 37% (95% CI 22–60%) (Figure 7).

### Mortality

**Figure 8:**
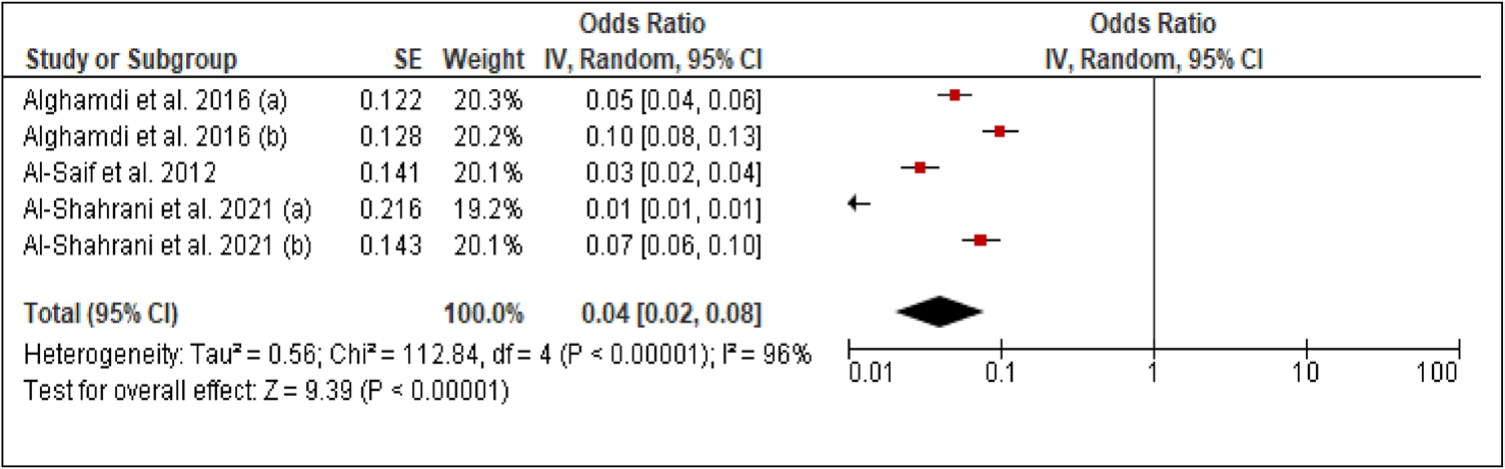
Forest plot of the prevalence of mortality among patients with PCAD. (**a**): under 45 years; (**b**): above 45 years.

Three studies reported the prevalence of mortality among patients with PCAD. The heterogeneity (Chi^2 ^= 112.84, *p *< 0.00001, I^2 ^= 96%) was high, so a random effect model was used. The forest plot analysis showed a pooled estimated prevalence of 4% (95% CI 2–8%) (Figure 8).

## Discussion

Coronary artery disease is a leading cause of morbidity and mortality worldwide. Premature coronary artery diseases have been correlated with multiple risk factors worldwide. Some of these factors are due to a sedentary lifestyle while some are genetically predisposed risk factors (modifiable and non-modifiable risk factors)^27–31^. Epidemiological data about Premature coronary artery diseases in Saudi Arabia are limited. To our surprise was the observation that although there is a long list of nontraditional risk factors significantly contributing to increased cardiovascular risk at younger ages, we did not find any report addressing the magnitude of these factors in PCAD. The list includes a group of factors that are categorized into lipid, inflammatory and prothrombotic markers.

To the best of our knowledge, this meta-analysis is the first to systematically identify several risk factors associated with PCAD, some are traditional while others are not, with a high prevalence of smoking, dyslipidemia, obesity, diabetes, and hypertension. There is a lack of studies correlating risk factors and PCAD in the Middle East. Therefore, we compared our findings with North American studies regarding PCADs.

Around 5–10 % of all acute myocardial infarction (AMI) cases occur in patients less than 45 years^32,33^. The percentages in Middle East countries are higher (11%) than in Western Europe (2.7%), North America (4%), and Africa (9%)^34,35^. Different types of smoking have been introduced and they are available in the market including traditional smoking, electronic smoking, and pipe (Shisha smoking). As a primary risk factor, smoking plays a major role in premature coronary atherosclerosis and in accelerating atherosclerosis by increasing the oxidation of low-density lipoprotein (LDL) and damaging coronary endothelial vasodilation^36^. In this context, many studies revealed that smoking was significantly associated with an increased risk of PCAD compared to a healthy population^37^.

In this meta-analysis, we analyzed 7 studies. The pooled estimated prevalence of smoking was 39% among Saudi patients, which is way lower than in USA patients with PCAD (60.8%)^38^. In addition, it is lower than what was reported in a study conducted in the United Arab Emirates, which reported a high prevalence of smoking associated with PCAD (70–90%)^39^. These dismal results need to be well evaluated by higher authorities to find solutions to this issue.

International Diabetic Federation (IDF) declared that the diabetes prevalence among Saudi adults is 17.6% which is considered one of the highest rates worldwide^40^. We found that the pooled estimated prevalence of diabetes among PCAD patients was 41% while diabetes prevalence in USA PCAD patients was lower (23.8%)^38^. Nevertheless, diabetes prevalence in PCAD patients was lower than in those with CAD (52–62%)^18,19,22^. It was reported that patients with diabetes have a two- to four-fold higher risk of developing coronary disease than people without diabetes^41^.

Indeed, diabetic patients exhibit an increased risk for the development of atherosclerotic CAD for many reasons, including metabolic factors, like hyperglycemia, dyslipidemia, and insulin resistance, which lead to endothelial cell, vascular smooth muscle dysfunction, impaired platelet function and abnormal coagulation^42^.

Our meta-analysis showed that the pooled estimated prevalence of hypertension in PCAD was 33% which is lower than the prevalence in the USA (52.8%)^38^ and the prevalence in CAD patients (36–68.7%)^18,19,22^. This could be due to arteriosclerosis (age-related disease) which increased the prevalence in older patients. High blood pressure can lead to CAD due to the added force on the artery walls. Over time, this can damage these blood vessels and lead to more plaque buildup. The narrowed artery limits or blocks the flow of blood to the heart muscle, which means it might not get enough oxygen^43^.

Based on two studies, the pooled estimated prevalence of overweight and obesity was 18%. In comparison to a study performed in the USA, they found that obesity (BMI > 30 kg/m^2^) prevalence in PCAD was 47.1% which is higher than what we found^38^.

In our study, the pooled estimated prevalence of a family history of CAD was 19%. In the USA, the prevalence was 39.8% which is higher than our findings^38^. This could be due to ethnic differences between the two populations. On the other hand, the prevalence of a family history of PCAD was higher than those with CAD (0.5–9.4%)^19,22^. These results highlight the importance of genetic and biomarker studies to understand the underlying mechanism.

Regarding dyslipidemia, the pooled estimated prevalence was around 37% which is lower than what was reported in the USA population (46.4%)^38^. These results suggest the need for extensive studies in predisposing genetic factors affecting lipid profiles. Nevertheless, lifestyle cannot be overlooked as an important contributor in this regard due to the high prevalence of physical inactivity and sedentary lifestyle in our region (overall prevalence of physical inactivity was 32.3%, and even higher in women 35.5%)^44^. Another systematic review in Saudi Arabia reported a worrying prevalence of physical inactivity in women ranging from 53.2 to 98.1%^45^.

The prevalence of drug abuse in PCAD was from 3.0 to 5.8%^21,22^. In a study performed in the USA detecting the correlation between Substance abuse and premature atherosclerotic cardiovascular diseases, they found a prevalence of alcohol was 31.8%, cocaine at 12.9%, amphetamine at 2.9%, and cannabis at 12.5%^46^. Thus, regarding amphetamine, our results are higher than those found in the USA which is a warning sign to be carefully considered.

Regarding mortality rate, the astonishing findings show that our results showed an estimated pooled prevalence of 4% (2–8%), which is similar to the prevalence in older patients (2–10%)^18–24^. This result should be the focus of extensive research, as well as legislators’ concerns.

In this regard, one cannot neglect the gender differences in risk factors in CAD or PCAD patients which need to be extensively evaluated. Last but not least, we found recently a high prevalence of lack of knowledge and unhealthy lifestyle practices of PCAD and its risk factors among the Saudi population where more intervention and awareness of health care providers to reduce its detrimental impact is of great value^47^.

### Strengths and limitations

In the present study, we performed a search in major databases. The main strong item of this article is the fair quality of the included studies. Although unpublished articles and local articles published in non-indexed journals have not been included in our study, the funnel plots did not show a publication bias. Furthermore, the main findings were validated by sensitivity analysis, which demonstrated the strong reliability of this meta-analysis.

One of the main difficulties is the large differences between studies in design, as well as in the cutoff point of risk factors. These differences present a significant limitation when comparing the results of different studies and consequently make pooled analysis more complicated. Hence, considerable heterogeneity, which is expected in meta-analysis studies, can alter the interpretability of results. Consequently, the findings of the present work must be analyzed with attentiveness. In addition, the lack of extensive and controlled studies was a limiting factor. Furthermore, a lack of studies concerning genetic differences as well as biomarkers affecting PCAD was also noticed. Also, all the eligible studies that have been included did not differentiate between males and females regarding risk factors that need to be considered in future studies. Also, none of the studies mentioned the occupation of the patients which we believe is an important risk factor that might contribute to premature coronary artery disease.

## Conclusions

The majority of the patients with premature coronary artery disease in Saudi Arabia are smokers, have dyslipidemia, diabetic and are overweight/obese. Smoking, diabetes mellitus, hypertension, family history of CAD, dyslipidemia, and overweight/obesity are significantly and positively associated with premature coronary artery diseases in Saudi Arabia. The other risk factors such as drug abuse and hypertriglyceridemia are associated with PCAD but to a lesser extent. We found an alarmingly high mortality rate of PCAD similar to CAD. The health authorities should design and implement an intensive and effective prophylactic plan to minimize the subsequent impact of PCAD on the young population. In addition, early diagnosis of PCAD has great value in providing timely treatment, managing the patients, and minimizing the burden of the disease.

### Supplementary Information

Supplementary Information.

## Data Availability

The data may be provided on reasonable request to the corresponding author.
